# Regulatory Integrity and Welfare in Horse Sport: A Constructively Critical Perspective

**DOI:** 10.3390/ani15131934

**Published:** 2025-06-30

**Authors:** Mette Uldahl, David J. Mellor

**Affiliations:** 1 Vejle Equine Practice, Fasanvej 12, 7120 Vejle Oest, Denmark; 2 Animal Welfare Science and Bioethics Centre, School of Veterinary Science, Massey University, Palmerston North 4442, New Zealand; d.j.mellor@massey.ac.nz

**Keywords:** equine welfare, equestrian governance, regulatory transparency, regulatory integrity, fit to compete protocols, coercive practices, behavioural indicators, tack and equipment, performance vs. welfare, horse agency, pain and fear in horses, evidence-based reform

## Abstract

This commentary evaluates contemporary equestrian sport governance through the lens of equine welfare science. Drawing on evidence from the FEI Sport Forum 2025 debates, the IFHA Racing Integrity Handbook, media coverage of horse sport, recent scientific presentations, regulatory texts, and published research, we identify systemic shortcomings in how horse welfare is assessed, prioritised, and protected. Key issues include reliance on performance as a proxy for welfare, inadequate “fit-to-compete” protocols, neglect of horses’ mental states, coercive tack and equipment practices (e.g., double bridles, tight nosebands, ear hoods), pharmacological and surgical interventions that mask pain, euphemistic regulatory language (e.g., whip “encouragement”), and inconsistent implementation of welfare rules. Through a series of case studies, from dressage and show jumping forums to racing integrity handbooks, we illustrate euphemistic language, defensive group dynamics, dismissive rhetoric towards evidence-based criticism, and a troubling “stable blindness” that sidelines the horse’s perspective. We conclude that meaningful reform requires (1) embedding validated behavioural and physical welfare indicators into all competition and pre-competition protocols, (2) transparent, evidence-inclusive rule-making under a precautionary principle, (3) genuine engagement with independent equine welfare experts, and (4) establishment of empowered, impartial oversight bodies to ensure that stated codes of conduct translate into consistent, enforceable practice. Only by catering to the horse’s subjective experiences and applying modern ethological and bioethical standards can equestrian sport retain its social licence and ensure integrity in all areas of competition management.

## Simple Summary

The constructive purposes of this commentary are to help secure a future for equestrianism by evaluating contemporary standards, identifying areas where the culture and structure of horse sport require modernisation, and suggesting solutions. It advocates incorporating the horse’s perspective and applying current, evidence-based knowledge in the training and use of horses. The article evaluates how horse welfare is managed in competitive equestrian sports. Despite growing scientific understanding of animal sentience and suffering, many traditional practices, such as the use of restrictive tack, pain-masking medication, and performance-based welfare assessments, still dominate. The article highlights the gap between modern welfare science and how horse sports are regulated, showing that many current rules do not protect horses from pain and fear. Using real-world examples, the authors argue that decision-makers in horse sport often resist change and dismiss scientific concerns. To ensure regulatory integrity and maintain public trust, the article calls for sports governing bodies to adopt clear, science-based welfare standards, involve independent experts, and put the horse’s experience at the centre of all decisions.

## 1. Introduction

### 1.1. Historical and Fundamental Perspectives

Equestrians of today inherited the customs, justifications, practices, and standards of the more experienced equestrians who trained them, who themselves had previously been trained by an earlier generation of equestrians [1]. A paramount dictum is that the equestrian is in charge, not the horse. Even if this is not stated, which usually it would be, it is obvious because horses do not volunteer to be trained and ridden or driven by equestrians [2]. Nor in individual cases, with rare exceptions when trained, would they choose to participate in specific competitive events such as gymkhanas, other horsemanship competitions, dressage, cross-country, show jumping, standard horse races, steeplechases, harness races, polo events, endurance races, and others [1].

Many of the most senior and highly regarded participants in, and regulatory guardians of, elite global equestrian competitions and overall horse sports learned about animal welfare when the science was in its infancy. This was when there was still scepticism about sentience in animals and their capacity to have negative experiences and when positive experiences were rarely, if ever, considered [3]. As a result, the constituents of suffering, including pain, fear, and panic, received little attention, and the signs of their presence were often overlooked.

### 1.2. Influences of Traditional Practices

The great antiquity of human–horse interactions means that human management of and attitudes towards horses have deep traditional roots. In the equestrian world, tradition is respected, so when horses are successful in competition, this is often attributed to tradition-based methods, which could involve cruel behaviour towards the horse [2]. Nevertheless, the comfortable familiarity of these methods, however noxious they might actually be, creates resistance to changes that are required to manage welfare concerns based on well-authenticated scientific findings (e.g., [4,5,6,7,8,9,10,11,12,13,14,15,16,17]). Resisted with equal vigour are suggested welfare-based changes to more recently modified practices or equipment that have traditional origins [1].

### 1.3. Resisting Needed Change

These and other challenges to what are common methods or practices (see later sections) cause great concern to elite equestrians and other horse sport professionals who rely on them to achieve and retain their competitive success. This brings with it a demonstrable desire to retain unchanged all facets of their training and practices that secured their influence, competitive status, and financial interests [18].

The resistance can manifest in various ways, including a reluctance to acknowledge potential compromises to equine welfare or the construction of alternative narratives designed to deflect public scrutiny. One illustrative example involves an elite rider attributing horses’ behaviour to “arrogance,” which the horse supposedly developed through awareness of competitive success when in the presence of other horses [19]. This statement epitomises anthropomorphism but may also represent an attempt to rationalise signs of frustration or distress observed in horses in elite competition environments.

When self-interest is threatened, this generates a strong desire to protect the status quo, where the status quo in these circumstances may well be taken to include ensuring that those affected can retain their current training biases [1,2].

### 1.4. The Sobering Impacts of the Drive for Needed Change

As noted above, the vast majority of those involved in horse sports and virtually every other equestrian activity, whether as participants or members of national or international standard-setting organisations, were taught traditional equestrian skills inherited from the past. However, as a wide range of standard practices and equipment previously used in good faith have now been challenged scientifically on welfare grounds (detailed in subsequent sections of this commentary), those previously unaware of these concerns may inadvertently have caused their horses’ welfare harm. However, as harm has now been demonstrated, the actions of equestrians who continue to use or the actions of standard-setting organisations that still require or allow the use of these practices or equipment are questionable.

Note that a key responsibility for directing change lies in the structures, culture, and decision-making of the standard-setting organisations [2], not the equestrians who follow the rules laid down by them. However, these organisations have historically and demonstrably shown reluctance to enforce existing welfare-related rules due to their potential impact on competition points allocations. Furthermore, they have also been hesitant to introduce and enforce additional measures to address other significant welfare concerns that remain unsolved [1]. Consequently, equestrians who wish to continue competing are compelled to adhere to the existing regulations, even when doing so will perpetuate welfare-related harm to their horses.

### 1.5. The Path Forward: Four Essential Components of Regulatory Improvement

Horse welfare is structurally compromised when organisers rate the success of competitions as spectacles and of the individuals participating in competitions ahead of the welfare cost to the horse. More specifically, this occurs when, for example, (1) outdated principles for handling and training are approved; (2) horse sports formats and equipment are adopted or retained despite bona fide welfare-based challenges to them; (3) the effective monitoring of horse welfare using validated (i.e., objective) behavioural signs of compromise is not in place; and (4) the well-authenticated, science-based principles and practice of horse welfare and its management, recognising the horse’s sentience and capacities to have negative and positive experiences, are not actioned. Attention to these matters would represent the minimum required to demonstrate that sport horse organisations are committed to taking their ethical responsibilities for horse welfare seriously.

However, as outlined in the following sections, standard-setting authorities are not only failing to meet these requirements but are also demonstrably resisting the implementation of necessary changes [1,2]. In some instances, this resistance has involved questionable tactics [20]. These developments raise important concerns regarding integrity, truthfulness, and trust, and the complex way in which these values intersect.

### 1.6. Integrity

#### 1.6.1. Personal Integrity and Its Relationship to Truthfulness and Trust

Integrity and trust are inseparable attributes, where the glue is truthfulness expressed as a commitment to the right action. Integrity also refers to the perceived reputational trustworthiness of individuals or members of institutions [21], such as members of national and international standard-setting organisations.

#### 1.6.2. Integrity and the Functioning of Organisations

When applied to organisations, integrity refers to the trustworthiness of their procedures, policies, priorities, and actions in support of achieving the values-based purposes for which they were created [21]. At their best, they would demonstrate that operationally they are admirably “fit for purpose,” a quality that could justifiably earn them considerable respect and recognition.

#### 1.6.3. Integrity and the Functioning of Whole Biological Organisms

This form of integrity indicates individuals’ capacity to maintain a healthy and balanced physiological state, supporting both physical and mental well-being. In the present context, this would be a horse as a holist, functionally integrated, sentient, autonomously living being, capable of enjoying good welfare states when given the opportunity [22].

### 1.7. Key Definitions

#### 1.7.1. Pain

In this paper, we adopt the International Association for the Study of Pain 2020 definition of pain as “An unpleasant sensory and emotional experience associated with, or resembling that associated with, actual or potential tissue damage” [23]. In line with the reasoning of Wilkins and colleagues in their 2025 submission to the FEI [24], the term pain is used to encompass experiences across a continuum from mild discomfort, which may signal the risk of tissue damage, to severe, excruciating pain, indicating significant or survival-threatening injury. Throughout this commentary, the entire spectrum of these experiences is referred to simply as pain.

#### 1.7.2. Fear

Like pain, fear is an evolved biological adaptation, a defence mechanism that serves to protect an organism against threats to its biological fitness, and is experienced as a negative emotional state [24]. While pain typically signals actual or potential tissue damage, fear is elicited by perceived threats to the body’s integrity and may arise even in the absence of physical harm [25].

#### 1.7.3. Pain and Fear: Closely Intertwined Responses

Thus, from an animal welfare perspective, pain and fear are closely interconnected, as fear represents a state where the animal anticipates harm and responds accordingly, for example, through avoidance behaviour. Importantly, from the animal’s perspective, the perception of threat, regardless of actual physical injury, can compromise welfare to a degree comparable to that caused by physical harm [26,27,28]. Accordingly, here, the term fear is used to encompass the full range of threat-related emotional experiences, from mild unease to intense fear.

### 1.8. Purpose and Contribution of This Commentary

The constructive purposes of this commentary are to help secure a future for equestrianism by evaluating contemporary standards, identifying areas where the culture and structure of horse sport require modernisation, and suggesting solutions. It advocates incorporating the horse’s perspective and applying current, evidence-based knowledge in the training and use of horses.

## 2. The Dual Aspect of Equine Fitness: Considering Both the Physical Health and the Mental State of Horses

When is a horse fit to meet sports competition requirements or those for racing? What parameters are available and could be implemented in regulations to manage the welfare of sports horses?

A longstanding view held by promotional and regulatory organisations for sports horse activities and horseracing is that the welfare of the horses can be gauged by their performance and physical health (e.g., [29]). This view is based on the notion that sports horses could only perform as well as they do if their overall welfare, including health, is first-class. There are several problems with this approach.

### 2.1. Performance as a Predictor of Horse Welfare

Horse sports disciplines have set formats where the aim is to entertain and impress onlookers, including judges. As an example, the equestrian purpose is to demonstrate rider mastery by making the horses perform in particular ways in competition with others. For riders not well versed in signs of compromised equine welfare, performance means the horse completes what is demanded of it in a timely manner, without apparent mishap, and with a precision that attracts high scores.

Common among such challenges are three-day events (eventing [30]). These consist of successive days of dressage, cross-country, and show jumping, where the same rider and horse compete throughout. Dressage involves horses often performing highly stylised, unusual movements in preset sequences lasting about eight minutes. Cross-country, performed against the clock, challenges both rider and horse as they negotiate a 2 to 6 km course over varied terrain. Depending on the competition level, the course includes 20 to 40 solid, challenging fences. It usually takes at least 10 min to complete the course. Showjumping, the final phase, also against the clock, involves 12 to 20 fences, some very high, others in challenging multiple sequences, with different shapes and contrasting colours, arranged in a complex pattern in a stadium. Occurring on the last day of the three, it is a serious test of the overall stamina and fitness of both the horse and rider. Some three-day-event commentators refer to the horse’s courage being seriously tested. However, as it is forced to take part, it more likely reflects the horse being able to display under duress skills-based tasks it was previously compelled to perform without the ability to refuse.

Competition riders, jockeys, and drivers possess exceptional skills in optimising equine performance to achieve peak results in competitions and races. However, striving for competitive success does not necessarily equate to prioritising horse welfare [18]. Current horse sport formats do not incorporate a direct, objective, or adequate assessment of equine welfare. This is reflected in there being weak indirect associations between judged competition success and negative equine welfare indicators [31,32], and in other research, more definitive negative correlations between judging scores and negative welfare parameters [33,34]; in other words, the higher the judged scores, the lower the welfare indicators.

In dressage, for example, judges frequently award high scores to horses displaying signs of pain, despite their official responsibility to penalise such behaviours [2]. This presumably occurs because objective assessments of equine welfare are not prioritised among the criteria required for judging successful horses. Accordingly, there is little incentive for those directly involved in horse sport to be informed and trained in contemporary evidence-based recognition of compromised equine welfare.

Also, it is well known from three-day events, racing, and research studies that horses, when pushed beyond their physical limits, can develop respiratory compromise [11,35,36,37,38,39] and fatigue, even exhaustion [37,40,41,42,43,44], which increase the risks of performance-related injuries, falls, and, in extreme cases, fatalities [45].

Clearly, simply performing a preset task successfully is not a reliable means of assessing the welfare of the horses involved.

### 2.2. How Effective Are “Health Assessments” as “Fit to Compete” Protocols?

Determining a horse’s fitness to compete or race has traditionally focused on pre-competition inspections, which typically include a veterinary assessment, albeit with varying levels of thoroughness.

Conflicting pressures are ever present during pre-competition inspections, particularly between the imperative to safeguard equine welfare and the desire of equestrians to compete. Thus, there is a key tension between the veterinary diagnosis of a health issue and the subsequent judgement of whether such a problem is sufficiently serious to warrant declaring the horse “unfit to compete.”

These pressures highlight the need to prepare and use detailed checklists of specific features to support each judgement concerning a horse’s “fitness” or “unfitness.” For such judgements to be credible, the criteria need to include features that are clearly based on a wider multidimensional understanding of equine welfare and soundness and not on one confined to a simple “health check.” Moreover, guidance on thresholds for acceptable states for each criterion needs to be provided. In the absence of such specificity, terms such as “fit” or “unfit” to compete, “sound” or “unsound,” or “accepted” or “not accepted” lack explanatory power and may drift in meaning over time, thereby becoming inconsistently applied by different groups of officials.

There is also the question of the expertise of those making these judgements and those who are qualified to make the final decisions (veterinarians or laypersons). In certain competitions, veterinarians serve solely in an advisory capacity, providing guidance to a panel of non-specialist officials on matters of equine health and soundness. However, the final decision rests with these officials, who may choose to override professional veterinary recommendations (FEI Veterinary Regulations 2025, article 1036, part 6 [46]). Given the substantial personal and financial interests at stake, it is imperative to uphold the principle of impartiality in the assessment of horses. To ensure objective decision-making, veterinarians and other relevant professionals should be appointed independently of the sport, competition, and organisations.

Although the paramount concern here is the welfare of each horse, such variability in decision-making is not in the interests of fair competition. In addition to an enhanced welfare focus, greater consistency and transparency of criteria used for each judgement would also result from implementing the above recommendations.

### 2.3. The Necessity to Evaluate the Mental State of Horses as Part of “Fit to Compete” Assessments

As noted in Section 2.1, equine performance in sport is neither a reliable nor a sufficient indicator of welfare. The physical and psychological pressure exerted by riders or drivers can override the horse’s natural limitations, potentially pushing the animals beyond their physiological and mental capacity. Such pressures may result in significant welfare compromises, adversely affecting the horse’s mental state. Additionally, Section 2.1 notes that current sports formats do not prioritise objective welfare assessment as a criterion for success.

While the traditional focus on the horses’ physical health offers some insight into their capacity to participate in competitions or races, this is contingent upon the level of scrutiny applied during pre-performance checks (see Section 2.2). However, developments in animal behavioural science have enabled identification of behavioural indicators that reflect a horse’s affective (emotional) state. These indicators, supported by extensive affective neuroscience observations in a range of species (see [47]), allow for a more nuanced assessment of whether a horse is experiencing a negative, neutral, or positive state. Integrating behavioural observations with physical assessments thus provides a more comprehensive evaluation of horse welfare, offering important insight into the horse’s mental states in different situations within and outside competition and training.

**Figure 1:**
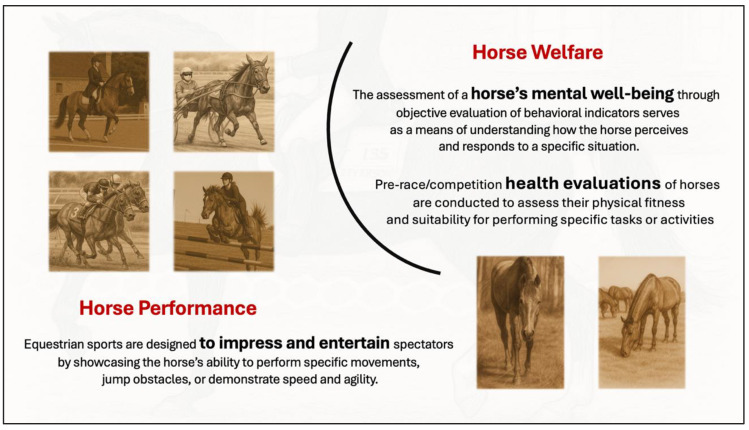
Judges’ scores, race times, clear jumping rounds, and performance in sport are different from assessing horse welfare (elements of illustration created using OpenAI DALL•E 3 model via ChatGPT; image: Shutterstock and Mette Uldahl).

Implementing objective assessments of the horse’s behaviour is required to advance contemporary horse sport towards modern welfare standards. As with physical assessment, the effectiveness of behavioural assessment depends on the quality and clarity of the protocols and their incorporation into sports formats. It is, therefore, important to ensure that evaluations are grounded in validated behavioural markers and that they are applied consistently. Moreover, the assessment of the horse’s mental state must carry a decisive weight in determining competition outcomes. If that is not implemented, there would be no formal curb on the welfare risks of win-at-all-costs demands on the horse (Figure 1). Examples of behaviours of known importance include mouth/lip movements, contraction of the eye, position of the ears, tail swishing, sudden head movements, and gait changes [15,33,48,49,50,51].

It is essential to ensure that the horse’s ability to express natural behaviours is not hindered by physical restraint or practices that conceal or suppress behavioural indicators.

## 3. Behavioural Control Through Tack and Equipment in Equestrian Practice

Viewed objectively and dispassionately, it is apparent that each item of tack was originally designed and used to impose the will of the rider or driver on the horse. This is the case for all types of tack, whether it is approved or not by horse sport regulators.

The use of all tack undoubtedly prevents the horse from fully exercising its “agency,” i.e., “the capacity of individual animals to engage in voluntary, self-generated, and goal-directed behaviour that they are motivated to perform” [52]. Note that such motivation relates both to seeking behavioural relief from negative experiences and to engaging in rewarding behaviours aligned with positive experiences [47]. Undermining either of these objectives carries adverse implications for animal welfare.

When competing or racing, it is usual for different configurations of tack to be fitted; first, to minimise the effects of individual “undesirable” behaviours [18,53,54], and second, to comply with event-specific behavioural requirements. The purposes are to prevent the horses from engaging in behaviours that would interfere with their performance or to change the horses’ behaviour and/or patterns of movement to improve the likelihood of competitive success. When faced with such tack-induced constraints, this would limit the horse’s natural drive to test a wide range of behavioural solutions to challenging situations [55], leaving resignation (i.e., submission) the only response available to it when “tacked up.”

In this construction lies a major risk for the horse to be treated merely as a piece of equipment that can be modified at the whim of the riders or drivers. Thus, whilst disregarding the welfare cost to the horse, they seek to coercively achieve the behavioural results required for them to be awarded high scores in competitions or to win races. The regulators claim that the welfare of the horse is their top priority, but judging by what they defend, permit, or require, it is apparent that many items of tack are used in coercive ways [1].

Safeguarding horse welfare necessitates granting horses a degree of agency: the ability to make choices and to avoid and resolve situations they perceive as aversive when they are motivated to do so [24]. This approach reflects a commitment to considering the horse’s perspective.

The industry involved in the development and production of tack and equipment for horses is substantial and is largely influenced by the tack regulations established by the sport horse organisations. The global equine equipment market was valued at USD 1975 million in 2020 (PR Newswire [56]). Given the significant influence this industry exerts over what products are developed and made available to customers, it is crucial for structural incentives within horse sport to promote the adoption of the least harmful and most welfare-focused equipment.

It is apparent that for tack-related reform to occur, attention needs to be refocused away from what equestrians want their horses to do towards what their horses experience as sentient creatures. In the context of tack and other restrictive equipment, it appears that their welfare-related experiences are predominantly negative [1].

## 4. Manipulation of Equine Physiology to Improve Human Competitive Advantage

There are numerous methods by which a horse’s ability to perceive stimuli or express their natural body language can be altered or compromised. Developed over the years, many of these practices are addressed by the regulatory measures referred to in the Sport Horse Regulations. Historically, attention in horse sport has primarily focused on the alteration of bodily functions through chemical means (e.g., medical doping), surgical interventions (such as neurectomy), or mechanical methods.

### 4.1. Pharmacological Concealment of Physical and Mental Impediments to Competitive Fitness: Selected Examples of Contemporary Relevance

Many research papers have documented discipline-related musculoskeletal problems in performance horses (e.g., [57,58,59,60]). These problems are often identified by behavioural signs of lameness, indicating pain [61]. Horses can develop these problems while competing and may continue to be entered in events despite the persistence of the problems. In some sports, it is common for horses to receive treatment to alleviate, for example, the pain from joint and tendon injuries [62,63], and in some cases, further treatment is permitted at events (e.g., FEIF General Rules & Regulations part G4.2 [64]; FEI veterinary regulations, chapter 5 Veterinary Medication [65]). For example, it is permitted for horses to be treated before the competition and while training when the drugs used have shorter retention times than the interval between the treatment and the competition (e.g., FEIF [64]; FEI [65]; UET Racing Animal Welfare Regulations [66]; NEMAC/DTC Racing Anti-Doping Regulations, §2 [67]). Also, some rules allow treatment at competitions (FEIF [64] General Rules & Regulations part G4.2, FEI [65] veterinary regulations, chapter 5 Veterinary Medication). Clearly, such allowable treatments could be used to mask the true “fitness” status of horses when presented for competition.

The high prevalence of stomach ulcers (Equine Gastric Ulcer Syndrome—EGUS) in sports horses is a cause for concern [68]. The major contributory factors are common management impositions that are related specifically to sports horse stabling, feeding practices, and the stresses of training, transport, and competition [68]. Avoidance or relief is achievable by changing these management practices to reduce the ulcerogenic challenge. However, the practices were originally devised to improve horses’ performances and/or their availability to compete [68]. It is no surprise, therefore, that continuous treatment with the efficacious pharmaceutical omeprazole is often preferred and commonly occurs. In effect, the pathophysiology may persist because its cause is not treated. However, horses retain the ability to compete because dosing with omeprazole reduces or eliminates most of the unpleasant sensations. Accordingly, omeprazole treatment effectively masks what would otherwise be a judgement of “unfit to compete.” Regulations relating to omeprazole are variable. It is not permitted by some authorities during competition and races (NEMAC/DTC Racing Anti-Doping Regulations [67]), yet others still allow it, even during competitions (e.g., FEIF General Rules & Regulations part G4.2 [64]; FEI veterinary regulations, chapter 5 Veterinary Medication [65]).

The use of certain medications may be permitted at a general level without requiring prior notification or approval from a governing body. This includes injectable treatments (e.g., vitamins, joint support therapies, amino acids, and injectable homeopathic remedies) as well as oral medications (e.g., omeprazole mentioned above and altrenogest, a synthetic progesterone to suppress oestrous in mares). Consequently, there is no official record of the overall number of horses treated with these substances during competitions or races, the frequency of injections given to an individual horse, or the veterinary justification for such treatments (FEIF [64]; FEI [65]).

Veterinary justification for the administration of medication presents another problem. Under European Union regulations, veterinarians are permitted to treat a horse only when a diagnosis of illness has been made and only in accordance with the conditions outlined in the medicine’s Summary of Products Characteristics (SPC) [69]. The SPC specifies the diseases, injuries, or deficiencies for which the product is approved (EU Regulation 2019/6, article 106.1). Consequently, treatment is only legally justified when a horse is diagnosed with a specified health condition that compromises its well-being.

Limited thus, this requirement appears inconsistent with the principle that horses participating in sport should be in optimal health and fully fit to compete. It also raises important questions about the appropriateness of administering medications registered for the treatment of illness and injuries in horses that are active in training and used for competitions or races, even at elite levels of competitions. If a horse is ill or injured, it should receive appropriate treatment and be allowed sufficient time for full recovery before being returned to sport.

Another category of substances in demand within equestrian sport is those that affect the horse’s mental state. Typically, they are calming agents. Although prescription anxiolytics or sedatives are generally prohibited, a grey zone exists involving presumed neuroactive substances that are not yet explicitly listed on prohibited substance registers but that are still administered with the intention of calming the horses. This category includes a wide array of over-the-counter products marketed for their calming effect. Some substances that were once commonly used for their calming effect have since been added to prohibited substance lists, such as alpha-casozepine [67,70].

A review of the regulations in this area reveals some confusion regarding their purposes. Regulatory organisations emphasise the importance of both fair play in competition and horse welfare, e.g., British Equestrian [71] and FEI [72] adhering to the Clean Sport principle led by the World Anti-Doping Agency [73], apparently conflating the justifications for both, whereas the two should be considered separately.

The Clean Sport orientation has the primary purpose of ensuring that all horses will compete on their own merits without a pharmacologically induced unfair advantage. For example, the Danish Equestrian Federation states in its regulations, part 23, that “Horses may not participate in equestrian events under the influence of painkillers, performance-enhancing substances, stimulants or sedatives” [74]. This explicitly prohibits direct interference with a horse’s mental or physical capacities by the use of such agents during competitions.

In this example, however, both purposes are served: fair play, as already explained, and avoiding welfare harms due to the potential for untoward drug-induced pathophysiological responses when the horse is exposed to the much greater demands of exercise while competing.

There is also the welfare question of why the calming agents are given in the first place. Is it primarily because the horses are exposed to greater stressors when transported to and from sports venues, while in unfamiliar venue stables, or when competing in strange arenas? Or is it because some horses are temperamentally more easily stressed than others? Or is it both? Clarification of such uncertainties would helpfully improve confidence in the reasoning given to support the intersecting objectives of competition, fair play, and safeguarding the welfare of the horses involved.

In conclusion, there is significant variation among horse sport governing bodies regarding the type of medication permitted for horses and their timing of administration relative to competitions or races. Notably, discrepancies also exist in the documentation and recording of medication use, which hinder the ability to accurately assess the scope of usage and its impact on horse welfare. In some cases, the administration of certain medications may serve to mask underlying welfare issues, which should be addressed through more responsible and sustainable structural interventions. Furthermore, existing regulations within the sport do not always align with current national or European Union legislation, raising additional concerns regarding legal coherence and enforcement.

### 4.2. The Use of Surgical Techniques to Improve Competitive Outcomes

Invasive (i.e., surgical) procedures performed on healthy animals have been classified as mutilations [75]. The justification for surgical interventions is questionable in animal welfare terms if their purpose is not to treat existing medical conditions or to prevent them [76]. This is particularly concerning when the explicit purpose is to permanently impair the functionality of the specific body parts, thereby covertly suppressing or obscuring behaviours that would otherwise result in lower competition scores.

For example, the nicking of tail tendons immobilises the tail and prevents the horse from exhibiting natural tail-swishing behaviour [77]. In addition to its fly-repellent purposes, tail swishing is among a horse’s behaviours that express its physical and psychological state, including the presence of stress and/or pain. Accordingly, in certain competitive disciplines, this behaviour is regarded as undesirable and may result in lower scores. A comparable unethical practice is the amputation of the tail [78], which is carried out for both traditional aesthetic reasons and to prevent the tail from becoming entangled in equipment. However, such risks could be effectively mitigated through less invasive means, such as bandaging or otherwise securing the tail hairs to prevent entrapment.

Likewise, amputation of the tongue [79] is an extreme intervention that is carried out to make a horse more competitive by preventing signs of stress and/or pain because a protruding tongue is a clear sign of bit-induced mouth pain [15]. In animal welfare terms, the immediate post-amputation consequences and early adjustments to the absence of a tongue are likely to be very unpleasant. Moreover, when eating, the tongue has major functional roles in chewing and swallowing, as well as in tasting different foods. The continuing daily deprivation or severe impairment of these functions would be exceptionally unpleasant, thereby magnifying the welfare harm to the horse.

Another practice involves surgically reducing the height of the spinous processes of the withers when a horse exceeds the 148 cm threshold used to define ponies in pony sport [80]. Such alterations are performed to qualify animals for a competitive category for which they would not otherwise be eligible (DEF Pony Measurement Regulation 2025, section 9; [81]). This practice is unacceptable both because it subjects the animal to an entirely unnecessary surgical procedure, potentially resulting in significant postoperative pain and protracted recovery, and because it is inherently deceptive in its intent.

Other surgical procedures are intended to mask chronic pain resulting from injuries or joint and tendon wear and tear. These include neurectomies of the foot [82] or suspensory ligaments [83], which desensitise the affected areas. Lesions requiring such procedures are often linked to discipline-specific, strenuous, or inappropriate training regimens [84,85,86] and thus would often reflect prior welfare compromise. Also, presenting horses to compete who have undergone such surgeries is intentionally dishonest and undermines principles of fair play. Clearly, this unscrupulously places such horses on a seemingly equal footing with those whose health and soundness have been preserved through responsible training and management.

Any surgical intervention that interferes with normal bodily functions or involves the removal of organs or body parts raises significant welfare concerns. These procedures carry a substantial risk of adverse outcomes, such as the development of neuromas, which can lead to long-term suffering [82,87].

**Figure 2:**
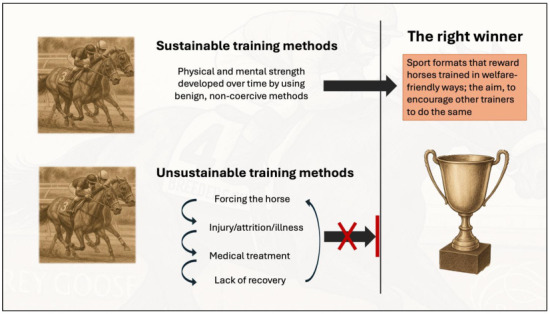
The prescribed format for a sport’s discipline significantly influences how horses are managed during training and competition. The right winners should be those managed benignly. (Elements of illustration created using OpenAI DALL•E model via ChatGPT; image: Shutterstock.)

Governing bodies in horse sport must remain fully aware of the potential financial incentives that may motivate trainers, riders, and owners to pursue such invasive measures. Vigilant regulation and proactive oversight are essential to ensure that competition horses are kept free of such welfare risks and the underhanded actions that cause them. The welfare guidelines from the International Group of Specialist Regulatory Veterinarians (IGSRV) act as an example of how this should be addressed openly [88]. Advocated here is the principle that the right winners should be the horses managed benignly (Figure 2).

### 4.3. Coercive Mechanical Practices and Methods Used to Manipulate, Dominate, or Force Horses to Perform in Particular Ways

A review of contemporary regulations reveals a history of practices being flagged, either for causing harm to horses or contravening the level playing field in competition.

Mechanical practices may include embedding sharp objects in leg protection (FEI boot and bandage tack control protocol 2025 [89]); rubbing the skin to increase sensitivity (FEI Veterinary Regulations 2025 [90]); or using techniques like “rapping” (FEI Jumping Rules [91]), all of which cause pain and merit banning on those welfare grounds alone.

Perhaps the most obvious mechanical device for coercive control of horses is the bit, where the controlling experience is the pain it causes or threatens to cause. Bit-induced mouth pain, its neurophysiology, affective neuroscience, pathophysiology, behavioural consequences, and major welfare implications have received considerable attention [11,15,24,92,93,94]. In this article, welfare issues associated with the use of bits are further examined in Section 4.3.2 and Section 5.1.2. The sobering implications of bit-induced pain are far-reaching and have been drawn to the attention of the FEI [24].

Additional examples of equipment or methods that have negative welfare impacts on horses include the following: over-tightening leg protection or bandages [89,90]; sharp objects inside leg protection [89,90]; heavy shoes, weighted boots, excessively long hooves [95,96]; overly tight nosebands [96]; over check [97]; draw reins [98]; suspensory devices in the vagina of a mare [99,100]; solid bars positioned on the side of the horse [97,99,100]; and other examples.

Examining all possible examples in detail is outside the scope of this commentary. Therefore, two representative cases have been selected to illustrate in greater depth the patterns of development and underlying objectives of certain practices. These examples highlight areas that warrant immediate attention and regulatory oversight by horse sport regulatory frameworks, particularly when there is an indication of compromise to horse welfare. They are: first, the neglected welfare compromise caused by the use of ear hoods and earplugs to manipulate auditory perception and restrict ear mobility; and second, the well-publicised continued practice of masking pain-induced oral behaviour by using tight nosebands to hide bit-related conflict behaviour.

#### 4.3.1. Use of Ear Hoods and Earplugs to Manipulate Auditory Perception

Hearing is a major element of the horse’s multimodal sensory capabilities, which also include sight, smell, skin sensations, and body positional awareness (proprioception). All of these are functionally integrated when the horse is in motion and are required, especially at speed and when jostling with other horses, as in races. Impairing any one of the major sensory elements would potentially be distressing for the horse, likely made worse in the often-disturbing contexts of competitions. To do so merely for competitive advantage cannot be justified, as it violates a major aim of animal welfare management, namely, the requirement to avoid actions that unnecessarily cause animals to have negative experiences [47,101].

The use of ear hoods and/or earplugs in both horse racing (parade ring) and equestrian sports activities (arena) has increased over the last 5–10 years [102,103,104,105]. Their use appears to have several purposes.

In some disciplines, like dressage, the main purpose is to impair the horse’s hearing [106], presumably to make the horse more tolerant of, i.e., less disturbed by, greater noise levels, patterns, and types during competitions, especially in enclosed arenas. In animal welfare terms, this has been viewed as a reasonable objective [106].

**Figure 3:**
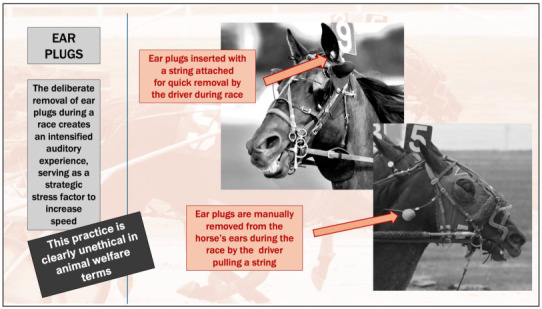
The earplug removal creates an intensified auditory experience, serving as a strategic stress factor to increase speed (Image: Shutterstock).

In other disciplines, like trotting, earplug/hood use is common from the beginning of the race until the earplugs are pulled out in the closing stages to boost the horse’s performance [97]. A specific example is when sudden earplug removal created a sound boost to the speed of “Commissioner King,” the Saudi Derby winner in 2023, where the faster pace enabled the horse to win the race [107]. The article reporting this [107] provided a comprehensive review of the effect of removing earplugs midrace. It clearly illustrates that stakeholders in horse sport are aware of horses’ highly sensitive auditory capacity as a prey species. The deliberate removal of earplugs is understood to create an intensified auditory experience, which acutely heightens the horse’s awareness of surrounding sounds and is strategically used to stress the horse to increase its speed and performance (Figure 3). This purposeful stressing of the horse to increase its speed demonstrates a callous disregard for the horse as a sentient being by deliberately violating its welfare state [47,101].

This example prompts critical questions about acceptable boundaries of auditory interventions. To what extent is it permissible to impose startling stimuli? Would placing a high-decibel loudspeaker near a horse’s ears ever be deemed acceptable? Conversely, in the case of sensory suppression, is it ethically justifiable to simultaneously restrict both auditory and visual inputs in a dressage horse prone to startle responses on the assumption that it would reduce its reactivity? In answer to this, it would first be necessary to demonstrate that any sense of vulnerability caused by impairing these two vital senses ‘would not, in fact, increase the horses’ startle responsiveness. Such an increase is plausible because, as a prey species, free-roaming horses, or members of their band on the lookout, continuously survey the surroundings, alert to threats and the need to escape by moving away, in extreme cases at a gallop [108].

#### 4.3.2. Use of Ear Hoods Restricts Ear Mobility

**Figure 4:**
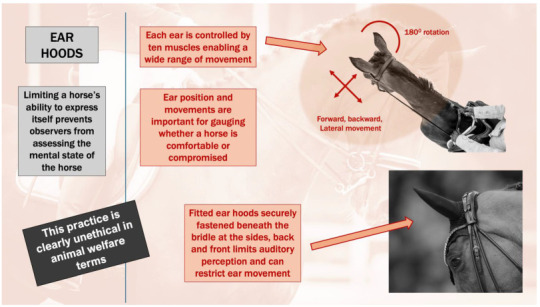
Earhoods lower auditory perception and limit the expression of earplay during competition, both of which are of ethical concern in governance of horses (Image: Shutterstock and Anders Deros/Aftonbladet).

The ears of a horse have a vital part in equine body language, serving as highly expressive organs that convey emotional states and behavioral intentions. Each ear is independently controlled by ten distinct muscles, enabling a wide range of movement, including backward, forward, lateral motion and rotations of up to 180 degrees [109]. This remarkable mobility allows horses to directionally perceive their auditory surroundings and communicate both with conspecifics and humans (Figure 4).

Ear position and movements are important signs for gauging whether a horse is comfortable or compromised. Many dressage horses are now routinely fitted with ear hoods; for example, 76% (13/17) of the participating horses in the FEI World Cup Final Grand Prix Freestyle 2025 [105] (see videos at ClipMyHorse). Fitted ear hoods, which are securely fastened beneath the bridle at the back and sides and held in place at the front by the browband, sometimes additionally tied to the noseband with a string, appear to restrict natural ear movement. This configuration tends to maintain the ears in a predominantly forward-facing position throughout judging sessions, in contrast to the greater mobility observed in horses not wearing hoods [104,105]. The neckpiece behind the ears can also be fitted very close to the ears or be very high, which will potentially also hinder free ear movements.

Ear movements are an important part of the horse’s body language and are an integral part of the “pain face” when that is present [110,111,112,113,114,115,116,117]. Accordingly, restricting these movements prevents riders, judges, spectators, and behavioural analysts from assessing related aspects of the mental state of the horse during competition. Also, depriving the horse of directionally focused reception of sound potentially compromises the horse’s ability to effectively interpret and respond to environmental stimuli within human-constructed settings (Figure 4).

The use of earhoods epitomises human-imposed interventions aimed at achieving specific objectives in equestrian sport by altering or restricting the horse’s natural sensory apparatus. Such practices raise ethical concerns because they undermine respect for the horse’s integrity as a holistic, functionally integrated, sentient, autonomously living being. Often justified in terms of “caring for the horse,” these interventions may, in fact, serve primarily to direct the horse’s participation towards human entertainment while subverting the animal’s autonomy and ability to give free expression to its own experience.

Finally, repeating two key points: first, ultimately, what matters to animals about their welfare is how they experience their life-world [47], and they need their full sensory apparatus to be able to do this unhindered; and second, equipment that conceals horses’ attempts to deal effectively with negative experiences for competitive advantage represents a breach of Clean Sport Principles [24].

#### 4.3.3. Well-Documented Concerns About Masking Oral Behaviour: Tight Nosebands, Bits and Related Horse Conflict Behaviours

One of the most common means of emphasising human dominance when using the bit and of camouflaging horse behaviour in competition is to use tightly cranked nosebands to prevent mouth opening and visible tongue movements, which, if observed, would incur penalty deductions of points [7,8,10,12]. It is important to understand that with such attempts to camouflage pain-related behaviours, using tight nosebands to mask the evidence of bit-induced pain does nothing to alleviate the mouth pain itself [118].

In fact, when cranked tight, nosebands themselves cause physiological signs of pain [7,10] and bone lesions [119]. The lesions indicate that the pain would be caused by mechanical stimulation of periosteal pain receptors due to localised high pressures of the noseband on bone [12,120,121]. In addition, tight noseband use causes oral lesions [13,49,122], which would also be painful [15].

Open mouth frequency and oral lesions indicate two different but related phenomena, both associated with pain. Frequent or persistent open-mouth behaviour is an immediate sign of acute bit-induced pain while being ridden [15], whereas oral lesions are due to injury of mouth tissues that have occurred previously and, as such, provide immediate and longer-lasting evidence of injury once riding sessions are complete [49].

Summing up, there are three sources of pain: bit-induced pain due to pressure on highly sensitive mouth parts; high noseband pressure causing periosteal pain on the nasal bone and mandible; and persistent pain associated with laceration and bruising of mouth parts caused during riding sessions.

Research has demonstrated that the prevalence of issues associated with tight nosebands and oral lesions is higher at elite-level competitions and is particularly pronounced in certain disciplines, notably dressage [13,122]. Furthermore, the incidence of oral lesions decreases by 34% when the noseband is loosened to allow for a space of more than 2 cm, with an additional 34% reduction observed when loosened to more than 3 cm [13].

The debate surrounding open mouths, tight nosebands, and oral lesions has been one of the most contentious within FEI equestrian sport. Riders and equestrian federations have generally adopted a very conservative stance, often resisting regulatory changes and repeatedly calling for additional research as a prerequisite for policy development. This is despite a substantial body of evidence based on decades of wide-ranging pain-related research in a range of animals [15] that consistently supports a clear association between tight nosebands, oral lesions, and open-mouth behaviours. Reluctance to accept these findings has been strikingly persistent [20].

Recently, a novel study comprising eight horses and five riders at the FEI level in dressage resulted in a publication examining aspects of pressure mat measurements in relation to noseband tightness [123]. In the study design, the noseband was adjusted to a maximum spacing of 2 finger-width equivalents or less, and the findings concluded no significant difference between 2 and 1.5 finger-width degrees of tightness. However, the study did not reference existing research that examined looser noseband fittings, specifically those exceeding more than 2 finger width equivalents [13], where the association with welfare concerns such as oral lesions was documented. Coinciding with the publication of this study, the FEI introduced a new regulation stipulating a permissible noseband tightness of 1.5 finger-width equivalents [124].

It is vital for the credibility and perceived reputational trustworthiness of a regulatory body to demonstrate that all relevant and valid evidence has been considered in the development of new rules or policies. In this context, the introduction of the new noseband regulation would have been strengthened by the publication of a transparent report detailing the sources of information and research that informed the decision-making process.

Such transparency not only enhances institutional trust but also enables informed public and stakeholder discourse on whether the rule aligns with contemporary expectations for equine welfare governance. Part of using research in policymaking is also to evaluate the outcomes in relation to the experimental designs. Underlying assumptions and conclusions reached must always be subjected to rigors evaluation as part of an overall review of existing literature.

An additional dimension of the ongoing debate surrounding oral behaviours associated with bit use concerns the use of double bridles in dressage. This practice has come under increasing public scrutiny, particularly in light of repeated observations at the elite level involving horses displaying open mouths and tongue discoloration—blue tongues [24,125]. These observations have prompted critical discussion regarding the necessity of mandating double bridles in competition and the professional justification for their continued use.

**Figure 5:**
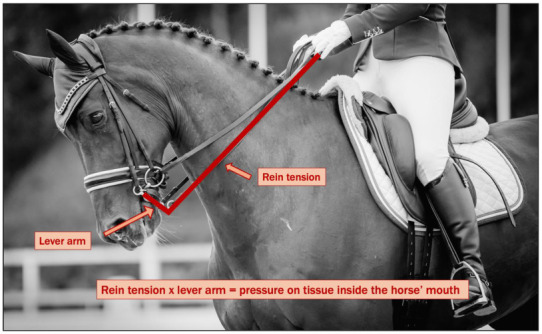
Measurement of rein tension is an example of why professional interpretation in relation to horse welfare is crucial: Rein tension between a rider’s hand and a lever of a bit measures the applied force on the lever. With leverage, the actual force on the horse’s soft tissue intraorally is markedly greater depending on the length of the lever. This understanding makes a great difference when comparing different types of bit (Image: Shutterstock).

A significant proportion of the criticism is related to the mechanical leverage exerted by the kerb (bridoon) bit and the overall volume for accommodating two bits within the horse’s mouth simultaneously. Leverage is known to amplify the force from the rider’s hands, thereby increasing pressure on sensitive oral structures, such as the commissures of the mouth, the bars, the tongue, and potentially the palate (Figure 5). For instance, a bit with a 1:4 leverage ratio means that one unit of pressure exerted by the rider’s hands on the reins translates to four units of pressure on the horse’s mouth [126].

A recent study investigated rein tension by measuring the force transmitted between the rider’s hand and the attachment point of the rein to the bit, comparing three configurations: a snaffle bit, a kerb bit, and a double bridle. No differences were found between the combined kerb and bridoon reins in the double bridle compared with tension in the snaffle rein. Additionally, when measured separately, both kerb and bridoon tension measured lower than the snaffle rein [127].

However, these results do not reflect the conditions within the horse’s oral cavity. Intraoral pressure is directly influenced by the mechanical leverage generated by the kerb shanks, which is not captured by external rein tension measurements. As a result, the absence of increased tension in the rider’s hand does not imply a reduced impact on the horse. On the contrary, lower rein tension in the presence of leverage may indicate that the horse is responding to a higher level of intra-oral pressure applied to highly sensitive oral tissues.

However, these outcomes do not reflect the conditions within the horse’s oral cavity. Intraoral pressure is directly influenced by the mechanical leverage generated by the kerb shanks, which is not captured by external rein tension measurements. As a result, the absence of increased tension in the rider’s hand does not imply a reduced impact on the horse. On the contrary, lower rein tension in the presence of leverage may indicate that the horse is responding to a higher level of intra-oral pressure applied to highly sensitive oral tissues.

For instance, the president of the Danish Equestrian Federation has referenced this study in a public debate regarding horse welfare to defend the use of the double bridle in sport, asserting that it generates less pressure than a snaffle bit [123]. This is an example of using research in a misleading way. It highlights the critical importance of ensuring that scientific research is interpreted accurately and within its investigatory context.

It underscores the need for comprehensive literature reviews conducted by independent and qualified professionals. This is needed to prevent misrepresentation or selective use of findings and when findings are inadequate to resolve specific horse welfare concerns so that erroneous interpretations are not used by horse sports organisations to shape policy or to influence public opinion.

### 4.4. Ensuring Horse Welfare: An Evaluative Approach to New Techniques, Equipment, and Practices

New techniques, medications, and equipment continue to emerge. It is vital to remain vigilant and to critically assess the intent and impact of such developments. Every piece of equipment and every practice serves a purpose, and changes in trends or usage patterns should be scrutinised with horse welfare as the primary concern.

A structurally methodical approach is essential, where asking key questions related to the intended function and providing justifications are mandatory. Key issues that must be examined are any potential to mask elements of domination of the horse, coercive practices, or other compromises to horse welfare.

In this context, the precautionary principle must be central: if any doubt arises against the welfare acceptability, safety, or other concerns regarding a particular method, practice, or piece of equipment, it should be prohibited until it can conclusively be demonstrated to not cause harm to the horse.

## 5. Evaluating the Underlying Motives of Governance Bodies When Their Stated Objectives Appear Disproportionately Amplified

Codes of conduct or practice and rules or guidelines issued by many sport horse organisations often contain broad statements of intent and responsibility [1]. Evaluating whether these stated objectives are effectively implemented in practice is best achieved by examining the existence and use of supporting protocols, the publicly expressed attitudes of individuals in leadership positions, prevailing practices, and the language used to frame contentious issues or practices in a more palatable manner [128,129,130].

It is not feasible to list or examine all relevant instances where the implementation of stated objectives appears questionable. Therefore, four representative cases have been selected. They provide opportunities to evaluate disjunctions between stated welfare positions and actual practice. The four examples are as follows:

- FEI code of Conduct versus a Sport Forum Discourse.
  ○ Example from Show Jumping;
  ○ Example from Dressage.
- IFHA Racing Integrity Handbook versus lack of implementation protocols.
- Presence of foam in the mouth obscuring oral evaluation during competition.
- Whip use regulations, deceptive terminology, and lack of conformity with equine learning theory.

### 5.1. FEI Code of Conduct Versus Sport Forum Discourse

Given the FEI’s key international leadership role, of particular importance is the example of its current Code of Conduct for the Welfare of the Horse [131]. It states that “at all times the welfare of the horse must be paramount” and “it must never be subordinated to competitive or commercial influences.” These opening statements are followed by more specific injunctions that include, but are not limited to, the following: stabling and feeding must demonstrate best practice; training must not be abusive or cause fear, nor should it use natural or artificial riding aids (such as whips or spurs); and only horses that are fit and competent in the sport can be permitted to compete. Importantly, regarding education, the Code states that the “FEI urges all those involved in equestrian sport to attain the highest possible levels of education in areas of expertise relevant to the care and management of the competition horse.”

#### 5.1.1. Example from Show Jumping

As an example of perspectives presented at the FEI Sport Forum 2025 [132], the issue of bleeding lesions on horses’ flanks caused by rider use of spurs was discussed. The debate emerged in the context of show-jumping riders opposing rules for them to be eliminated when such lesions were detected. The president of the International Jumping Riders Club remarked that “a scratch and elimination is unfair,” emphasising that these injuries are “not done on purpose.” In response to the view that non-purposefully inflicted bleeding scratches would be acceptable, a director of a National Equestrian Federation questioned, “How are we going to explain it to the public?” The chair of the FEI Jumping Committee further noted, “…we are living in times where it is very difficult to explain to the outside and inside world” [132]. This example highlights a significant disregard for horse welfare, as well as riders’ lack of awareness of, or unwillingness to accept, their role in contributing to the lesions observed on the horses. Furthermore, it reveals that the governing body, alert to rising public concerns about sport horse welfare, was apparently focusing more attention on public perception than on a genuine concern for the harm done to the horses.

#### 5.1.2. Example from Dressage

In the discussions at the FEI Sport Forum 2025 [132], the use of double bridles and their potential to inflict oral pain, including whether discoloration of tongues is seen as problematic towards horse welfare or not, were vigorously debated. Both forum attendees and members of the newly formed Dressage Strategic Action Plan Working Group expressed reluctance to address these welfare concerns. As Lise Berg (Ass. Prof. of Applied Clinical Biomedical Science, Copenhagen University) stated, “Evidence and evidence-based, has become weaponised.” Similarly, elite trainer and rider Kyra Kyrklund noted that while dressage was once criticised “as a bit boring” and was thus made more spectacular, it has “now gone over the line” and must be scaled back. Later, she challenged the view that going behind the vertical with regard to the horse’s jowl angle should be condemned as abuse and characterised public and social media critics as “almost religious sects” whose “chief priests” are not to be reasoned with.

Throughout the debate, critics from outside the dressage community were repeatedly said to “attack” the discipline. FEI Dressage Director Ronan Murphy insisted the sport faces “issues” rather than “systemic problems,” a view echoed by Kyrklund. Jason Brautigam, CEO of British Dressage, queried whether there is a consensus on hyperflexion (low jowl angle) and tongue discoloration (hypoxia or blue tongues) because when judging, “we don’t see a problem” … “so how do we educate the general public?” [132].

Overall, these statements suggest that the defenders of dressage implicitly acknowledge the credibility, authority, and evidentiary strength of the science presented—science that is detailed, factual, and produced by internationally recognised experts. The defenders’ inability to offer a coherent rebuttal underscores the absence of a cogent defence.

Accordingly, their behaviour in response appears to reflect the following features:

(1) A sense of helplessness in the face of robust and compelling evidence.
(2) An ostensibly self-protective scepticism towards the evidence, often accompanied by calls for further proof as a means of delaying action.
(3) A related reluctance to acknowledge welfare compromises specific to dressage, including their behavioural and physical manifestations.
(4) A tendency to characterise the presentation of scientific findings as unfounded “attacks” or as examples of “weaponised” science.
(5) In the absence of valid scientific counterarguments, efforts are made to discredit the scientists themselves, branding them as “close minded zealots” or likening them to “chief priests” within so-called “religious sects.”
(6) An emphasis on reassuring the public that dressage practices are unproblematic despite credible concerns raised by leading international experts.

**Figure 6:**
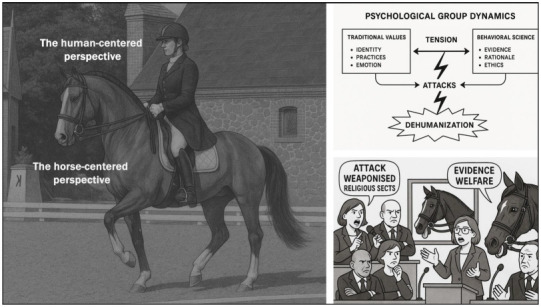
The expression of group dynamics in horse welfare debates often reflects the tension between communities with deep-rooted identities, traditions, and practices and the evolving ethical expectations informed by contemporary animal behavioural and welfare sciences (elements of illustration created using OpenAI DALL•E model via ChatGPT, Image: Mette Uldahl).

Such defensive, emotion-led, and, at times, aggressive rhetoric is characteristic of group dynamics, wherein challenges to traditional practices are perceived as an assault rather than constructive feedback. Such conflicts typically escalate from mild disagreement to outright dismissal, often denigrating and dehumanising opponents [133,134] by labelling them as “critics,” “outsiders,” “activists,” “non-riders, who don’t understand the reality,” and the like (Figure 6).

At the same forum, the FEI Veterinary Director stated, “we are engaging with some scientific groups, as well as others from the opposite direction to ensure a constructive dialogue” [132]. About two months before the forum, a coalition of scientists, including one of this article’s co-authors (David Mellor), had submitted a letter of concern to the FEI regarding thousands of photos from recent FEI Horse Shows, documenting significant welfare issues associated with the double bridle. A subsequent 50 min presentation to the FEI Veterinary Committee [24] detailed oral injuries, tongue compression, tongue discoloration, pain-related behaviours, and respiratory distress concerns, which, at the earlier Sport Forum, had been rhetorically dismissed.

This stark divergence in welfare perspectives is central to the broader debate in equestrian sport. A performance-centric view risks overlooking mild to severe welfare compromises. Conversely, a horse-centred approach, grounded in objective behavioural and physical markers, reveals a fundamentally different reality. Accordingly, horse sport must engage with equine experts together with, repeat, “together with” traditional performance stakeholders. Otherwise, a narrow, sport-focused “stable blindness” may prevail, and modern welfare standards informed by contemporary equine science will be neglected.

### 5.2. IFHA Racing Integrity Handbook Versus Lack of Implementation Protocols

The International Federation of Horseracing Authorities (IFHA) Racing Integrity Handbook [135] emphasises the centrality of horse welfare in racing, stating that horses must be prioritised throughout their lifecycle, with welfare extending beyond minimum standards to encompass a “life worth living.” It stresses the need to protect horses not only from injury and death but also from factors negatively impacting their quality of life, asserting that performance should never compromise welfare.

The handbook outlines key welfare principles, including zero tolerance for cruelty, the importance of regular care, injury prevention, and humane euthanasia. It references the Five Domains Model, particularly Domain 5, which addresses horses’ mental and emotional states, emphasising factors such as comfort, social bonding, and human–animal relationships.

A chapter on equine behaviour highlights the need for space and appropriate facilities to enable natural behaviour but provides limited guidance on behavioural assessment. It recommends monitoring mental indicators such as alertness, engagement, and the absence of anxiety or fear, yet lacks specificity.

The trainer’s obligations focus on nutrition, veterinary care, and care of injured horses, but again, objective assessment protocols are absent. The IFHA Minimum Horse Welfare Guidelines 2023 [136] call for preventing “unnecessary” pain and distress, although the term “necessary” remains undefined despite the extensive scientific literature on equine pain and distress.

The role of racing authorities in safeguarding horse welfare is addressed only broadly, and steward qualifications include just one equine-specific criterion: knowledge of a horse’s condition and temperament. This reflects a broader issue: the lack of detailed, validated standards and objective assessment methods for ensuring equine welfare.

Overall, the IFHA Handbook and Guidelines provide general welfare principles but insufficient detail for consistent, science-based implementation. As a result, welfare practices, particularly regarding horses’ mental states, are left to subjective interpretation rather than grounded in validated behavioural science.

### 5.3. Presence of Foam in the Mouth Obscuring Oral Evaluation During Competitions

For digestive purposes, horses produce 20–40 litres of saliva each day [137]. Under normal circumstances, this saliva is swallowed and is not noticeable. However, any obstruction of its passage from the salivary glands within the oral cavity to the stomach via the oesophagus can disrupt this process.

A commonly recognised example among horse owners is oesophageal obstruction, often referred to as choke, which is a frequent equine emergency typically caused by feed material blocking the oesophagus. One of the symptoms is profuse salivation, and it necessitates immediate veterinary treatment [138]. In addition to mechanical obstructions, excessive salivation may occur in response to local irritants affecting the oral mucosa. It can vary in severity depending on the underlying cause [139].

When horses are ridden, varying amounts of foam may be observed around the edges of the lips, commonly referred to as “lipstick.” This comes from the high concentrations of the surfactant protein latherin, which helps the horse to digest dry forage. Latherin is also present in horse sweat and has a natural tendency to foam with friction. As such, foaming may occur when a horse mouths the bit or simply moves the mouth without a bit. Foaming is predominantly related to exercise involving human interaction. A small amount of foam around the edges of the lips is generally accepted as a positive sign, but the context and relation to welfare have not been researched.

The accepted degree of foam sometimes referred to as “happy foam,” has been questioned [140]. However, it is known that more or excessive foam can indicate significant compromises. In the context of dressage, the present authors have identified four factors that can impede normal swallowing of saliva during dressage sessions: (1) the persistently open mouth; (2) immobilising the segment of the tongue deep in the throat, proximal to the bit; (3) a consequent inability or markedly reduced ability to swallow, also noted by others [10]; and (4) the required vertical or near-vertical orientation of the head and the very low jowl angle. Accordingly, any saliva produced can only leave the oral cavity via the horse’s mouth. It is also possible that the bit as a foreign body in the mouth might increase saliva production [92,140], but the 4-point explanation holds whether saliva production increases or not. This analysis challenges the assumption that foaming indicates equine comfort or contentment, instead suggesting it reflects a state of significant pain and associated distress.

Assessment of the oral cavity, including the frequency of mouth opening and the colour of the tongue, has become an integral component of behavioural and physical evaluations of horses in competition. This reflects the growing application of animal behavioural science in various settings to assess equine welfare.

Advances during the past decade in our understanding of behavioural and physical indicators of equine mental state and compromised welfare have made this possible. These developments have been further supported by improvements in the quality of photographic and video documentation.

In this new era, where it is possible to get deeper insight into the horse’s experience during ridden exercise, it has become increasingly clear that welfare concerns do exist. Detailed reports have documented tongue discoloration in elite-level dressage [24,125], as well as high frequencies of pain-induced mouth-opening behaviour associated with oral lesions detected after the dressage session [49]. Public interest in these issues has led to criticism of the discipline, with individual horse/rider combinations as examples.

In recent years, there has been a noticeable change in the appearance of oral foam in some elite-level dressage horses during competitions. This shift pertains not only to the quantity of the foam but also to its structure and stickiness, which differ from what was commonly seen before.

As a result, the oral cavity is increasingly obscured, preventing thorough visual assessment by officials, judges, media representatives, and animal behavioural analysts. The mucosa of the tongue and oral cavity is partially or even fully covered by this dense foam, which can obstruct the evaluation, for example, of the colour of the tongue, an important indicator of hypoxia (i.e., a bluish discolouration).

In 2023, FEI implemented a ban on the use of artificial foaming agents [141], implicitly acknowledging that this practice was known to have occurred. At the 2025 FEI World Cup in Switzerland, it was reported that officials instructed that the horses’ mouths be wiped free of foam before entering the arena [142]. This may have been intended to reduce the visual obstruction caused by potentially excessive foam, thereby facilitating welfare assessments, or to present a more conventional appearance to the public, thereby mitigating concerns about whether oral function is abnormal.

**Figure 7:**
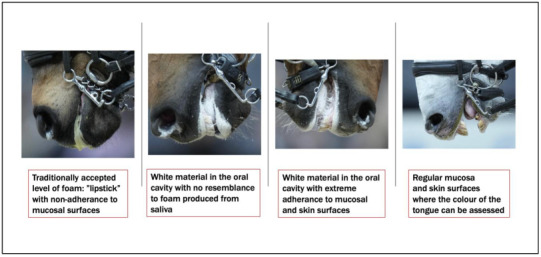
Following the emergence of discussions regarding open mouth and tongue discoloration (blue tongues) in dressage horses, an increasing trend of excessive and artificial-looking foam was observed. Such practices raise ethical concerns both due to pain experienced by the horse and the apparent intent to limit observers’ ability to detect the signs of pain (Photo credit: Anders Deros/Aftonbladet).

A 2025 newspaper article brought attention to the concerning practice observed at an FEI World Cup dressage event. Several veterinarians, including one of the authors of this article (Mette Uldahl), reported that photographs taken at the event depicted horses with a sticky white substance adhering to the mucosal and skin surfaces of their mouths and covering their tongues. This material, which, according to the veterinarians, clearly differed from the regular type of foam, is sometimes produced during riding (Figure 7). They expressed concern that event stewards failed to take appropriate action in accordance with the rules. Despite these observations, the event organiser reported no deviations from the norm, and the FEI informed the newspaper that it does not consider the photographs to constitute evidence of a rule violation [143].

This situation illustrates the implementation of a rule without a clearly defined enforcement protocol. Several critical questions arise (Figure 7). What constitutes artificial foam? Does this definition include any substance given to the horse at the time of bridling? Is a specific timeframe prior to competition stipulated?

These uncertainties highlight the need for explicit and well-defined regulatory frameworks to effectively address emerging practices and trends. Without it, it is not possible to apply sanctions in cases where a horse displays abnormal foaming patterns, so the intended purpose of the rule may be undermined, rendering it ineffective in practice.

In theory, a rule that cannot be effectively enforced may be perceived as a symbolic gesture, even a ruse intended to create the appearance of action while the action is not taken. This concern was echoed by the Danish Animal Ethics Committee in their 2023 report on the use of horses [144], in which they noted that while many regulations contain well-intended provisions, there is a pressing need for greater alignment between stated intentions and practical implementation [145].

Accountability, transparency, and reliability in rule enforcement are important for securing a social licence to operate. Failure to align policies with these principles risks eroding public trust and the perceived integrity of the governing body [146].

### 5.4. Whip Use Regulations, Deceptive Terminology, and Lack of Conformity with Equine Learning Theory

Whip use has been a controversial matter of welfare concern for some time. Driven by the likelihood that members of the public would regard whip use as cruel, horseracing authorities have devised diversionary euphemisms for describing its purposes.

For example, New Zealand Thoroughbred Racing (NZTR) [147] frames whip use in terms of “integrity,” stating that it serves to “encourage due effort from the horse if used when in winning contention or achieving a stakes’ bearing position.” Integrity in this context appears to mean that racegoers can rely on the horses to run without stints. Likewise, the British Horseracing Authority (BHA) [148] asserts that “The use of the whip in British racing is restricted to safety and encouragement. By “encouragement,” we mean using the whip as an aid to activate and focus the horse so the horse realises its potential by giving its best. Use of the whip to coerce is not permitted, and the rules are designed to reflect this.” These statements appear to be constructed with betting stakeholders and the general public in mind, aiming to reassure audiences by distinguishing between “encouragement” and “coercion.”

Since 2022, Danish trotting has adopted the term “animation” [97,99]. Within this regulation, the whip is referred to as a tool for communication and correction of the horse, deemed an essential element of safety equipment.

Euphemistic terminology is also prevalent in broader equestrian sports. The Danish Equestrian Federation (DEF General Regulations, part 16) [149], under the section “Riding Technique and Effect in Training and Competition,” stipulates that “the whip may only be used to guide and correct the horse in accordance with the horse’s natural behavioral reactions and learning potential. It must never be used as punishment.” This description implies that the whip functions as an extension of the rider’s arm, akin to a pointing device, rather than as an instrument to strike a horse. However, in contrast to this general statement, the discipline-specific rules for jumping explicitly permit a horse to be whipped twice “per incident” (DEF Jumping Rules, based broadly on the FEI Jumping Rules) [145]. Although it is noted that the whip may not be used as punishment but only for correction or reinforcement purposes, the distinction remains ambiguous. The FEI addresses this in its show jumping rules (FEI Jumping Rules 2025) [150], which state that a horse must not be hit more than three times consecutively.

Several concerns arise from these formulations regarding whip use. First, the application of euphemistic language and unsupported assumptions, such as the whip being an essential safety tool, can be interpreted as rhetorical tactics, a form of welfare washing. From the horse’s perspective, such semantic distinctions are unlikely to be meaningful. Painful or aversive experiences are processed in similar ways regardless of the terminology used to describe them.

Second, there is a notable absence of clear guidance or protocols outlining specific contexts in which whipping a horse is deemed appropriate. Vague descriptors such as “incidents” (DEF Jumping Rules) [145] or subjective phrases like “animation should always be ethically appropriate and may not put unnatural pressure on the horse” [97,99] lack precision and invite misinterpretation.

Thirdly, these regulatory frameworks lack alignment with established principles of learning theory. For example, the assertion that the whip may not be used for punishment is conceptually inconsistent with behavioural science. Any application of a whip that causes pain constitutes an aversive stimulus. Here is where distinguishing between “correction” and “punishment” becomes a semantic exercise rather than a meaningful behavioural distinction. It is not plausible to suggest that horses are capable of distinguishing between these human-imposed categories. If the intended distinction relates instead to the degree, duration, or context of whip use, such parameters should be explicitly defined in clear, unambiguous language to prevent misinterpretation and misuse.

Ultimately, the use of rhetorical strategies to reframe whip use risks eroding public trust in regulatory authorities. As these tactics become more widely recognised, they may contribute to diminishing the credibility of standard-setting organisations, i.e., their perceived integrity, and thereby jeopardise public support for horse sport [. This poses a severe threat to the sector’s Social Licence to Operate [151].

World Horse Welfare [WHW] has provided an extensively referenced review on whip use and five of its conclusions are as follows [152]:

1. There is no proof that the use of the whip makes horses race faster or slow down less;
2. There is no evidence that use of the whip improves steering, reduces interference, or increases safety;
3. Use of the whip has been associated with decreased safety;
4. Although there may be no definitive proof that whipping causes pain in horses, there is likewise no proof that it does not cause pain, and every reason to believe that it does;
5. World Horse Welfare and the RSPCA, who advise the British Horseracing Authority (BHA) on welfare issues, do not support the use of the whip for “encouragement.”

The second author of this commentary (David Mellor) has spent at least 30 years researching the pain caused by routine injury-inducing husbandry practices and the alleviation of that pain in a range of livestock species [118]. Based on that experience, it is apparent that the WHW’s fourth conclusion reflects a degree of caution or scepticism about whether animals can, in fact, experience injury-induced pain, which is at least 30 years out-of-date [153]. As stated briefly, as sentient animals, there is no doubt that horses can and do feel pain when whipped, i.e., when the whip is applied firmly [154].

### 5.5. Welfare in Name Only: The Credibility Gap Between Welfare Rhetoric and Reality in Horse Sport Governance

Thus, in light of the above sections and as concluded in published studies, it is simply not credible to claim that equine welfare is a high, or even the highest, priority for sport horse organisations [1]. This view agrees with an earlier report by the Danish Animal Ethics Council (2023) [144]. Its members expressed serious concern about the use of horses in sport, noting that although regulatory statements regarding their welfare were already included in the code of conduct and the rules, there was little evidence of actions taken to put them into effect.

Likewise, the notion that sport horse welfare is free of “competitive or commercial influences” is implausible [1]. As already referred to above, these are clearly the primary motivations for the understandable resistance of elite equestrians to accept bona fide welfare-related recommendations that would challenge their current success by changing their training and riding practices.

Part of the solution to secure horse welfare in sports lies in governing bodies living up to their stated objectives. For example, FEI living up to the very last statements of its code under the heading of Education [FEI Code] [131], namely, that “This Code of Conduct for the Welfare of the Horse may be modified from time to time and the views of all are welcomed. Particular attention will be paid to new research findings and the FEI encourages further funding and support for welfare studies.”

## 6. Integrity in Horse Sport Governance

As the entities responsible for formulating and enforcing the rules that directly affect horse welfare and equestrians, decision-makers within horse sports organisations should conscientiously acquire an in-depth understanding of the foundational animal welfare sciences developed over at least the last three decades [3]. They should also seek to know how those sciences underpin contemporary welfare-related research currently being applied to horses (e.g., [118]).

Open-minded consultation with a broad and credible spectrum of professionals who possess expertise in relevant areas will be required, as well as a preparedness to accept that some conclusions will be challenging or uncomfortable. The accuracy of judgements made by horse sports organisations regarding the depth, breadth, and quality of research findings presented to them will depend on how well they have understood the array of sciences that underlie contemporary animal welfare knowledge, especially equine welfare. Moreover, any claims of evidence-based decision-making must involve a transparent and thorough consideration of all relevant scientific publications. All credible results, investigations, and observations should be systematically and transparently included in the decision-making process.

The reputational integrity of such organisations will depend on the extent to which their revised and new regulations transparently reflect evidence-based knowledge and contemporary welfare standards. Crucially, it will also depend on the clarity of the criteria used to demonstrate that all regulations are actioned to good effect and are not merely window dressing. Consistent with this, all decisions must place the horse’s perspective and its welfare at the forefront, above human preferences or competitive interests [3].

When they are both rule-makers and enforcers, such sport horse organisations have a clear responsibility to ensure that the welfare standards they adopt are based on in-depth, objective, and rigorously supported scientific knowledge so that those standards are defensible in a modern ethical context [3]. An absence of such rigour and clarity would undermine the credibility of their publicised welfare-based decisions and statements of intent and would damage their reputational integrity. This, in turn, would weaken their capacity to fulfil another of their acknowledged roles, namely, securing the continuation of horse sports by constructively responding to such challenges as those enumerated above. Confidence that sport horse welfare is well understood and maintained will be essential for the retention of public support, i.e., this sector’s Social Licence to Operate [151].

The Danish Animal Ethics Council issued a statement in 2023 regarding the use of horses in sports in Denmark (DAEC 2023) [144], which should alert Danish and other national organisations to the need for more effective follow-through. As noted above, the report outlined current practices and regulations in relation to the governance of horse welfare, and one of its key conclusions, based on independent evaluations, was that while codes of conduct and regulations contain many well-intentioned principles, their practical implementation remains insufficient.

The documented continuation of contentious practices related to inadequate rules highlights a fundamental issue: human-focused participants in horse sport, or others with any relation to the sport, cannot always be relied upon to prioritise horse welfare, particularly when competitive success is at stake. Independent input must, therefore, be guaranteed.

A potential solution would be to establish independent advisory boards for horse sports with authority to issue guidance that horse sports organisations are obligated to follow (e.g., [8,155]). Such a structure would help ensure that decision-making remains scientifically informed, ethically sound, and genuinely focused on equine welfare.

An independent assessment of horse welfare and the governance system was recently conducted by inviting the welfare group R-Haltenswert to the FEI World Cup Finals 2025 in Switzerland. Prior to the event, the show organisers had stated their commitment to “a zero-tolerance approach to misconduct,” with particular emphasis on actions that compromise horse welfare. It was reported that several issues were identified [156], underscoring the value of independent evaluations, as these may offer perspectives on horse welfare that differ from those of stakeholders embedded within the sport. Such assessments invariably foster relevant discussions that should remain strictly objective, with the horse’s perspective serving as a starting point [3].

## 7. Discussion

Incorporating animals into competitive sports introduces a range of ethical and welfare challenges, particularly due to the complex psychological dynamics that characterise human competitive drives; the human urge to win at all costs, coupled with the idolising of athlete figures as heroes, can lead to the glorification of attendant equine suffering. Also, some riders, jockeys, or drivers anthropomorphize their horses, describing them as being proud of their performance or even exhibiting arrogance, which reflects human projections rather than equine experiences.

Although many riders profess to prioritise horse welfare alongside performance, the pressures of competition often reveal a shift in priorities, with success taking precedence.

This tendency is well-documented in sports psychology, where the pursuit of recognition and victory frequently overrides ethical considerations.

If regulatory frameworks governing horse welfare in sport fail to account for these inherent aspects of human behaviour, they risk being ineffective in safeguarding animal welfare. Structural welfare concerns manifest across disciplines, regardless of specific demands, whether it be speed, gait, gymnastic precision, or jumping abilities. Despite differences in external presentation, the core dynamics compromising equine welfare remain strikingly similar.

Pre-competition assessment of horses is a key example. Without detailed and standardised protocols, decisions regarding fitness to compete may vary significantly. If evaluators lack experience or the necessary expertise in equine physical, physiological, behavioural, and mental health, there is a heightened risk of unfit horses being allowed to compete, leading to negative welfare outcomes.

Likewise, the regulation of permitted tack and equipment must be guided by objective, evidence-based assessment. Permitted devices should be regularly reviewed in light of contemporary scientific knowledge concerning associated risks of negative impacts on equine welfare.

The manipulation of horses’ bodies for competitive advantage must be subject to rigorous scrutiny. Practices such as surgical alterations (mutilations) that inhibit expression and responses related to sensory perception or analgesic interventions that mask pain without addressing underlying issues represent a significant concern. The normalisation of such interventions, often justified under the guise of maintaining performance, undermines the fundamental principle, here emphasised on animal welfare grounds, that only healthy, well-conditioned horses should be eligible for training and competition.

Medication policies in sports must be critically examined as the definition of doping varies across equestrian disciplines. Usually, it is merely a list that each sport horse organisation has decided upon themselves with variable, arbitrary, often inadequate consideration for the horses’ welfare. The administration of substances solely to enable participation, particularly when rest or changes in training practices might be more appropriate, raises serious ethical questions. Horses should only receive medical treatment in response to a diagnosed illness or injury, accompanied by a plan for restoration, rehabilitation, and evaluation of training practices.

The use of mechanical devices to coerce performance, especially those that induce pain or force unnatural postures, must always be assessed from the horse’s perspective. Training methods should always adhere to the principles of equine learning theory. Such methods should also prioritise safety, understanding, and the least aversive approaches, whether or not the alternative approach is more time-consuming or otherwise challenging for humans to implement. If a horse appears incapable of performing a task, it should never be forced to comply.

Interventions that impair sensory function, such as the physical obstruction of vision (blinkers) or hearing (earplugs), are indefensible from an animal welfare perspective. The perceived necessity of such measures signals underlying structural issues within the discipline that must be addressed directly. For example, in relation to sound, the use of amplified, blaring music or loud microphone announcements during performances or in the vicinity of stable areas may contribute significantly to sensory overload and stress.

Euphemistic language remains a pervasive issue in horse sport, often obscuring harmful practices under the pretence of protecting the horse. Painful or restrictive interventions should never be misrepresented as protective. Transparent, objective terminology must replace such deceptive language to maintain public trust and ethical clarity, the hallmarks of reputational integrity.

When equestrian organisations claim to base policies on scientific evidence, it is essential that the evidence incorporate comprehensive literature reviews. Selective citation, or exclusion of relevant studies, risks accusations of bias and undermines credibility in the decision-making process.

These issues are critically relevant to the long-term sustainability of horse sport, which must not be secured through trade-offs that compromise horse welfare. Accordingly, the internal culture must evolve to genuinely prioritise horse welfare in both policy and practice. Governing bodies must put in place explicit measures to circumvent any tactics designed to delay the implementation of improved welfare standards and/or to weaken any existing high welfare standards.

To ensure transparency and accountability, it is recommended that governing bodies adopt arms-length principles, engage independent assessors, and establish advisory boards comprising experts in animal welfare sciences and veterinary medicine. The appointment process for such bodies must remain independent of the sport’s internal structures, and their mandate should focus solely on the horse’s perspective. Their recommendations, particularly when welfare concerns are raised, must be acted upon as a matter of obligation, not discretion.

## Data Availability

Not applicable.
